# Optical, Structural, and Crystal Defects Characterizations of Dip Synthesized (Fe-Ni) Co-Doped ZnO Thin Films

**DOI:** 10.3390/ma13071737

**Published:** 2020-04-08

**Authors:** Ahmad M. Alsaad, Ahmad A. Ahmad, Qais M. Al-Bataineh, Areen A. Bani-Salameh, Hadeel S. Abdullah, Issam A. Qattan, Zaid M. Albataineh, Ahmad D. Telfah

**Affiliations:** 1 Department of Physical Sciences, Jordan University of Science & Technology, P.O. Box 3030, Irbid 22110, Jordan; sema_just@yahoo.com (A.A.A.); qalbataineh@ymail.com (Q.M.A.-B.); areensalameh92@gmail.com (A.A.B.-S.); abdullahhadeel@yahoo.com (H.S.A.); 2 Department of Physics, Khalifa University of Science and Technology, P.O. Box 127788, Abu Dhabi 127788, UAE; issam.qattan@ku.ac.ae; 3 Department of Electronic Engineering, Yarmouk University, Irbid 21163, Jordan; zaid.bataineh@yu.edu.jo; 4 Leibniz Institut für Analytische Wissenschaften-ISAS-e.V., Bunsen-Kirchhoff-Straße 11, 44139 Dortmund, Germany; telfah.ahmad@isas.de; 5 Hamdi Mango Center for Scientific Research (HMCSR), the Jordan University, Amman 11942, Jordan

**Keywords:** optical properties, structural properties, crystal defects, dip coating technique, (Fe-Ni) co-doped ZnO thin films

## Abstract

Sol-gel technique is used to synthesize as-grown zinc oxide (ZnO) and iron-nickel (Fe-Ni) co-doped ZnO thin films deposited on glass substrates using dip coating technique. The structural properties and crystal imperfections of as-prepared thin films are investigated. We performed the structural analysis of films using X-ray diffraction (XRD). The XRD analysis reveal that the as-prepared films exhibit wurtzite structure. Furthermore, XRD-line profile analysis is performed to study the correlation between structural properties and imperfections of the nanocomposite thin films. The crystallite size and microstrains parameters are predicted using the Williamson–Hall method. We found that the crystallites size increases as the co-doped (Fe-Ni) concentration is increased. However, microstrains of the nanocomposite films decreases as (Fe-Ni) concentration is increased. The optical properties of the (Fe-Ni) co-doped nanocomposite films are investigated by performing UV-Vis (250 nm–700 nm) spectrophotometer measurements. We found that as the (Fe-Ni) concentration level is steadily increased, transmittance of the undoped ZnO thin films is decreased. Remarkably, refractive index of undoped ZnO thin films is found to exhibit values extending from 1.55 to1.88 that would increase as (Fe-Ni) concentration is increased.

## 1. Introduction

Semiconductor conducting thin films have gained a lot of momentum owing to their physical and chemical properties. They have proven to be practical, easy to process, and cost-efficient contenders for the synthesis of primary components for the industry of electronic, piezoelectric, and optoelectronic devices for current and next generations. Translucent oxides such as CuO, SnO_2_, and ZnO have invaded photovoltaic and detecting systems industrial sectors [1,2]. The inherited high excitation binding energy of ZnO [3], physical and chemical stability [4]. Based on these observed outstanding properties, ZnO has been widely used in the fabrication of photodetectors, reminiscence uses, LED, solar cell, gas devices, and acoustic wave detectors [5,6]. Controlling the preparation conditions, in particular, annealing temperature is crucial for obtaining high-quality thin films. Several methods and techniques were used to prepare the undoped and doped ZnO films such spray pyrolysis [7], successive ionic layer adsorption and reaction (SILAR) [8], magnetron sputtering [9], sol-gel spin coating [10], pulsed laser deposition [11], and atomic layer deposition [1]. 

Transition elements like Fe, Co, and Ni possess outstanding physical properties and huge magnetic moments essential for the fabrication of spintronic-based and optoelectronic devices [12,13]. Because of the partially filled *d* states of transition metals, they exhibit magnetic behavior vital for spintronic-based quantum computers. The research works that reported the optical properties of Fe, Co, and Ni-doped ZnO are scarce. For the fabrication of quantum well devices for particular applications, band altering property plays a critical role. Band gap of ZnO can also change by Fe, Co, and Ni doping. Furthermore, *3d* partially filled *d* states in Fe, Co, and Ni contain unpaired electrons accountable for displaying magnetic behavior. The insertion of *3d* transition metal ions such as Fe and Ni into ZnO matrix can shift the Fermi level that results in engineering the band gap [12,13,14]. 

Fe-doped ZnO and Ni-doped ZnO thin films have different physical, chemical, structural, and optical properties than the corresponding properties of ZnO thin films [12,13,14,15,16,17]. Iron and nickel atoms have high atomic numbers (Z of Fe = 26 and Z of Ni = 28) respectively. Fe^+2^ and Ni^+2^ ions have large atomic radii, 1.56 Å and 1.62 Å, respectively. Furthermore, the electron work functions of Fe and Ni are 4.50 eV and 5.01 eV, respectively. The large and close values of work functions of both ions make them eligible and appropriate as co-dopants in ZnO thin films. Moreover, Fe and Ni exhibit high first ionization energies of 7.902 and 7.639 eV, respectively. Thus, both can substitute Zn in the wurtzite hexagonal network of ZnO structure. To elucidate the appropriateness of Fe and Ni as co-dopants in ZnO, we compare their lattice and electronic properties with the corresponding values of Zn atom which exhibits Z = 30, ionic radius of Zn^+2^ = 0.23 Å, electron work function of 4.28 eV and first ionization energy of 9.394 eV. We may realize that either of the two doping elements (Fe or Ni) would substitute Zn atom in the hexagonal platform [18,19]. Likewise, the electron affinity of Fe is ≈4.50 eV and that of Ni is ≈5.01 eV. Both values are larger than the corresponding value of Zn atom which exhibits no stable negative ion state. This implies that both Fe and Ni atoms have greater deal of affinity with O atom compared to that of Zn atom during (Fe-Ni) co-doped ZnO thin film growth. Ni atom has greater affinity with oxygen than iron has, gives it the priority of substituting Zn with O. Hence, deformed oxides appear in thin films, distorting their physical properties.

We focus on optimizing the experimental conditions in order to produce doped ZnO films appropriate for particular applications, such as photovoltaic cells, solar cells, or optical coatings. Remarkably, we are able to accomplish this target by co-doping the ZnO films with adjustable Fe and Ni content ratios to achieve the required characteristics for specified electronic, magnetic, and optoelectronic devices. What makes this kind of co-doping very peculiar is that, by adjusting the Fe/Ni ratio, diluted magnetic semiconductors (DMS) based on ZnFe_1−x_Ni_x_O (x≤0.1) nanorods could be successfully synthesized by simple preparation techniques such as hydrothermal method. The microstructure, morphology, optical, and magnetic properties of DMS can be characterized using simple XRD, UV-Vis, and SEM techniques. 

The physical and optical properties of the undoped ZnO and (Fe-Ni) co-doped ZnO thin films are the main themes of our current research. In particular, structural properties of thin films such as lattice constants, lattice volume, crystallites size, micro strain, dislocation density, and other crystal imperfections are investigated. Furthermore, optical properties such as complex refractive index, complex dielectric functions, and optical band gap energy will be studied and interpreted. Up to the best of our knowledge, we are not aware of any experimental study that has previously investigated (Fe-Ni) co-doped ZnO thin films for optical applications.

## 2. Experimental Details

### 2.1. Undoped ZnO Solution Preparation

In order to prepare ZnO solution, we dissolve 4.38 g of purified zinc acetate dehydrated (Zn (CH_3_CO_2_)_2_·2H_2_O) in 50 mL absolute ethanol (99.85%) to get 0.4 M volume concentration. The prepared ZnO solution was shaken homogenously using a magnetic stirrer for 50 min at 25 °C until it turns milky. Obtaining creamy solution is an indication of mixing the solution ingredients homogenously. To obtain a transparent solution, a volume of 1.7 mL ethanolamine stabilizer was poured into the solution dropwise while stirring. The final mixed solution was stirred for 40 minutes and clarified by paper-filter with 0.45 μm in dimension [20,21]. 

### 2.2. (Fe-Ni) Co-Doped ZnO Solution Preparation

In order to prepare Fe-Ni co-doped ZnO solution, 2, 4, and 8 wt.% of iron (III) nitrate nonahydrate (Fe (NO_3_)_3_·9H_2_O) and nickel (II) nitrate hexahydrate (Ni (NO_3_)_2_·6H_2_O) were poured into ZnO solution in order to prepare final solution of Fe-Ni co-doped ZnO films. The ratio Fe/Ni was kept to 1:1 to maintain an equal concentrations of (Fe:Ni) co-dopants. The entire solution was shaken thoroughly on a magnetic stirrer for 30 min at room temperature until it become homogeneous and filtered by paper-filter with 0.45 μm in dimension.

### 2.3. Undoped ZnO and (Fe-Ni) Co-Doped ZnO Thin Films Fabrication

ZnO and Fe-Ni co-doped ZnO solution were then deposited via dip coating technique for 2 h on pre-cleaned glass substrates. The films were dehydrated in oven under atmospheric pressure for 15 min at 110 °C in order to evaporate the solvents and organic deposits. Owing to our previous experience with ZnO thin films, we annealed undoped ZnO and Fe-Ni co-doped ZnO films in air at 500 °C for 2 h to optimize their structural parameters [20,21].

## 3. Results and Discussion

### 3.1. X-ray Diffraction (XRD) Analysis

#### 3.1.1. ZnO and (Fe-Ni) Co-Doped ZnO Thin Films Structure Analysis

**Figure 1:**
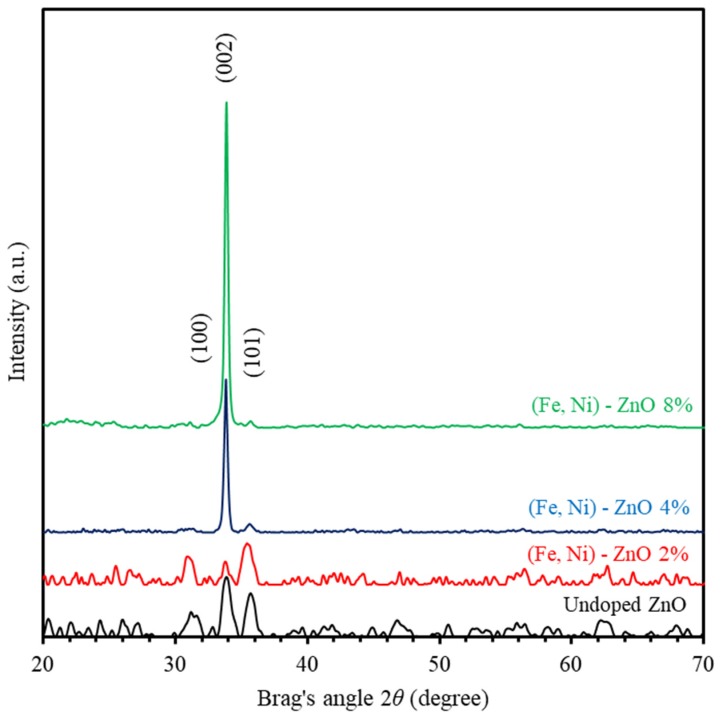
The X-ray diffraction (XRD) patterns of ZnO and Fe-Ni co-doped ZnO thin films.

XRD (Malvern Panalytical Ltd, Malvern, UK) is used to study the crystalline nature of undoped ZnO and (Fe-Ni) co-doped ZnO thin films. Figure 1 shows the XRD patterns of ZnO and (Fe-Ni) co-doped ZnO thin films annealed at 500 °C for 2 h. The main peaks of undoped ZnO thin films are observed at Bragg’s angles of 31.31°, 33.87°, and 35.69°, corresponding to ZnO crystallographic planes indexed by Miller indices of (100), (002), and (101), respectively. Comparison of the observed and standard “d” values (hkl) planes as matched with JCPDS cards no. 036-1451 and 071-3830, indicates that ZnO thin film is polycrystalline and exhibit wurtzite hexagonal structure [22,23]. Inspection of the XRD patterns of (Fe-Ni) co-doped ZnO thin films grown with 4% and 8% (Fe-Ni) concentration levels show that (002) crystallographic orientation attains the highest intensity. This is due to the grain growth associated with (002) preferred orientation and/or increase in the degree of crystallinity upon introducing 4% and 8% Fe-Ni in ZnO thin films. Moreover, Figure 1 shows a slight shift of all diffraction peaks toward the lower diffraction angles (2θ) as (Fe-Ni) concentration level increases. In order to get a deeper insight into the diffusion of different species in the interstitial slots of ZnO thin films, we calculate the atomic and ionic radii of Zn^2+^, Fe^2+^, and Ni^2+^ ions and found them to be 1.42 Å, 1.35 Å, 1.40 Å, 1.06 Å, 1.35 Å, and 0.83, respectively. Subsequently, it is easy to figure out that upon introducing Fe-Ni ions in ZnO thin films, Zn^2+^ and Fe^2+^ ions diffuse in the interstitial spaces between Zn^2+^ and O^2−^ ions leading to the shift of the positions of XRD peaks. 

**Figure 2:**
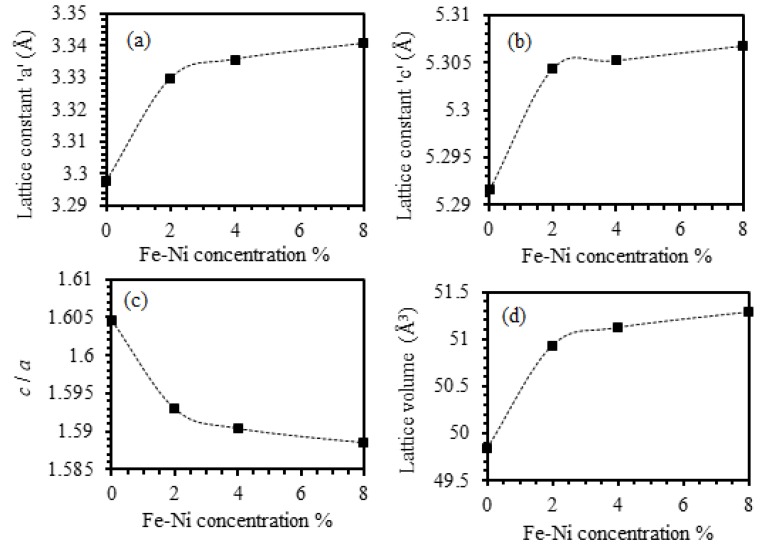
Lattice constants (a and c), the ratio of (c/a) and the lattice volume (V) determined from X-ray diffraction pattern of the undoped ZnO and the Fe-Ni co-doped ZnO thin films as a function of (Fe-Ni) concentration. (**a**) In-plane lattice constant a as a function of (Fe-Ni) % concentration. (**b**) Out-of-plane lattice constant c as a function of (Fe-Ni) % concentration. (**c**) Axial ratio (*c*/*a*) as a function of (Fe-Ni) % concentration. (**d**) Lattice volume (V) as a function of (Fe-Ni) % concentration.

Further analysis of the XRD patterns indicate that pure ZnO thin film with hexagonal structure and space group C6v4-P63mc exhibit in-plane and out-plane constants of a=3.2489 Å and c=5.2068 Å as indicated by JCPDS cards no. 036-1451 and 071-3830 [22,23]. The lattice constants “a” and “c” of wurtzite structure are given by $a=\lambda /\sqrt{3}\sin\theta_{\left(100\right)}$ and $c=\lambda /\sin\theta_{\left(002\right)}$ [24,25,26,27,28,29], where λ is the wavelength of the X-ray (0.1540598 nm) and θ is the angle of incidence [30]. In addition, lattice volume (V) of a unit cell of Fe-Ni co-doped ZnO thin films is given by $V=\left(\sqrt{3}/2\right)a^{2}c$ [24]. The calculated values of the lattice constants (a and c), the axial ratio (c/a) and lattice volume as a function of dopant concentrations are displayed in Figure 2. We found a and c of undoped ZnO thin film to be 3.298 Å and 5.292 Å, respectively, which is in good agreement with the standard values recorded on JCPDS cards no. 036-1451 and 071-3830 [22,23]. Careful examination of a and c of 2%, 4%, and 8% Fe-Ni co-doped ZnO thin films indicate that films exhibit (a and c) values of (3.329 Å and 5.304 Å), (3.336 Å and 5.301 Å) and (3.341 Å and 5.307 Å), respectively. Our results show that, introducing Fe-Ni modifies the lattice parameters slightly. Furthermore, the intensity of the peak corresponding to (002) crystallographic orientation is large enough to show that this orientation is preferred by undoped ZnO, 4% and 8% Fe-Ni co-doped ZnO thin films. In addition, we found that ZnO films co-doped with 2% of (Fe-Ni) exhibit (101) crystallographic orientation. Although the value of *a* and *c* increase by increasing the Fe-Ni concentration (Figure 2a,b), the axial ratio c/a was found to slightly decrease from 1.605 for the undoped ZnO film to 1.589 for doped films. The decrease in the value of *c/a* is small to be adequately treated as constant. Similar behavior is adopted by ZnO thin films co-doped with different concentrations of (Fe-Ni) as demonstrated in Figure 2c. In addition, we found that V increases, while the axial ratio *c/a* exhibits a slight decline as Fe-Ni concentration level is increased, as clearly illustrated by Figure 2c,d. Remarkably, we found that shrinkage in V is almost isotropic indicating that by applying mechanical stress along a given direction, a strain is produced along different direction of the crystal. Our findings imply that, the Fe-Ni co-doped ZnO thin films exhibit extraordinary characteristics enabling them to fulfil all requirements for promising piezoelectric applications, which will be addressed in the following section. 

#### 3.1.2. The Crystallite Size and Microstrain Analysis

**Figure 3:**
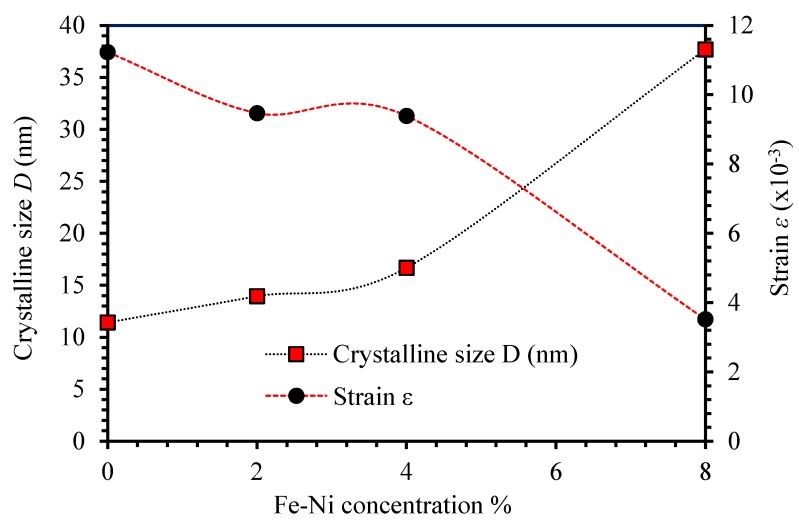
The crystallite size, D and the microstrain 〈ε〉 of undoped ZnO and Fe-Ni co-doped ZnO thin films as a function of their concentration.

For a crystal to become perfect, it has to expand to infinity in all dimensions. Such expansion produces XRD line-patterns rather than XRD peaks. Therefore, no crystal is ideally perfect because of its finite size that causes the XRD line-patterns to broaden and produce peaks rather than lines [31]. The crystallite size and lattice strain are the main characteristics extracted from the peak width, intensity and shift the 2*θ* peak position. Crystallite size is the size of the coherently diffracting domains. The formation of polycrystalline combinations makes the crystallite size of the particles different from the particle size [32]. The average crystalline size D can be calculated from Debye Scherrer’s formula D=kλ/βcosθ [24,33], where D is the crystallite size, λ is the wavelength of the X-ray (λ=0.154184 nm), β is the full width at half maximum (FWHM) after correcting for instrumental peak broadening (β expressed in radians), θ is Bragg’s angle and k is Scherrer constant. The value of k depends on the shape of the crystal, the diffraction line indices, and the dispersion of the crystalline sizes of the powder [34,35]. Usually, k is typically between 0.8 and 1.39 and for the spherical particles k is nearly 0.94. Lattice strain or micro strain is a measure of the distribution of lattice constants arising from crystal imperfections, such as lattice dislocations, the grain boundary triple junction, contact or sinter stresses, stacking faults, and coherency stresses [36]. The peak broadening is a result of micro strains occurring because of the displaced atoms that are rearranged with respect to their referenced lattice-points and because of the lattice defects occurring throughout the domain [37]. The micro strain is defined by the changes in the d-spacing throughout the domains which depends on the material elastic constants and the kind of internal stresses. The micro strain 〈ε〉 of the thin films can be expressed as 〈ε〉=βcotθ/4 [33]. The experimental conditions associated with the deposition process such as thermal and kinetic energies of the molecules on the substrate cause random crystal growth. This leads to an internal residual stress (tension or compression) that leads to the deformations during the film crystallization. The origin of the micro strain 〈ε〉 is related to the lattice misfitting and mismatching [38]. The estimated values of the crystallite size, D and the average micro strain 〈ε〉 were plotted as functions of Fe-Ni concentrations as shown in Figure 3. The crystallite size of ZnO thin film was found to be 11.43 nm. However, introducing Fe-Ni as co-dopants to ZnO films was found to increase the crystallite size to 13.95 nm, 16.69 nm, and 37.69 nm for 2%, 4%, and 8% Fe-Ni ZnO thin films, respectively. Moreover, increasing the (Fe-Ni) co-doping level was found to increase the crystallite size and improves the degree of crystallinity. This could be attributed to the fact that insertion of Fe-Ni into the unit cell of ZnO causes the cell volume to expand and thus causes strong attraction force between Fe-Ni ions with O^−2^ ions and strong repulsive forces with Zn^+2^. The electron affinity of Zn^+2^ ions is negative (−50 kJ/M) while that of Fe^+2^ and Ni^+2^ are positives (+15.7 kJ/M and +112 kJ/M, respectively) indicating that Zn^+2^ is more likely to combine with O^-2^ compared to Fe-Ni ions. The combined attraction-repulsive interactions enhance the diffusivity of the oxygen and zinc ions forming deformation in the domains that enhances the di-electricity of the material, and hence enlarges the ZnO unit cells. It was shown that additional doping of Fe-Ni stimulates the cells buckling [39,40]. This causes atoms to accumulate, leading to an increase in the crystallite size. Our results agree well with the previous findings [39,40,41]. Interestingly, co-doping of more Fe-Ni into ZnO thin films leads to a decrease in the values of local micro strains. The observed converse relationship between the micro strain and the crystallite size is due to the decrease in the volume occupied by the confined atoms inside joint crystalline structure. We found that increasing the volume of the unit cells leads to an increase in the total surface area that causes the shift in planes positions [42].

#### 3.3.3. Dislocation Density and Crystalline Density

**Figure 4:**
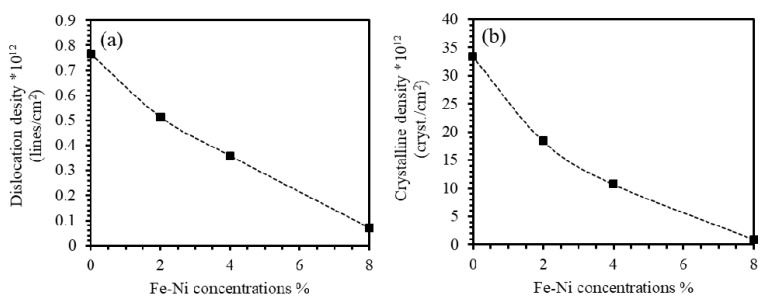
(**a**) Dislocation density and (**b**) crystalline density of undoped ZnO and Fe-Ni co-doped ZnO thin films as a function of Fe-Ni concentration.

Dislocations are the most important crystal defects that may occur due to internal stresses. The linear imperfections acting as boundaries between the slipped and the un-slipped regions occur in the slipped planes and called dislocations. The generated dislocations react by a driving movement when stress is applied on the domain. The dislocations play a crucial role in determining the strength and ductility of materials. [43]. We calculated the density of dislocations (δ) by using the line profile analysis of X-ray diffractions (LPA-XRD) and using simple Williamson–Smallman formula given by $\delta =1/D^{2}$ [33,43,44,45,46], where D is the value of the crystallite size. Figure 4a shows the dislocation density of the undoped ZnO film and the Fe-Ni co-doped ZnO thin films as a function of Fe-Ni concentrations. The value of (δ) of ZnO thin films was found to be $0.77\times 10^{12}$ lines/cm^2^ that decreases by introducing Fe-Ni co-dopants in ZnO films as clearly seen in Figure 4a. The value of (δ) characterizes the crystalline clusters-spaces and their agglomerations. A decreasing (δ) value implies AN enhancement of the crystallization and diminishing of the free spaces. It also implies a reduction in the interstitial vacancies by adding more of Fe-Ni into ZnO thin films [43,45].

The crystalline density (*N*) of ZnO Fe-Ni co-doped ZnO thin films can be calculated from the estimated values of crystallite size (*D*) and given by $N=t/D^{3}$ [47,48], where *t* is the film thickness found to be 500 nm. Figure 4b shows the crystalline density of undoped ZnO and Fe-Ni co-doped ZnO thin films as a function of Fe-Ni concentration. The value of (*N*) of ZnO thin films was found to be $0.33\times 10^{12}$ cryst./cm^2^. This value was decreased to $0.18\times 10^{12}$ cryst./cm^2^, $0.11\times 10^{12}$ cryst./cm^2^, and $0.09\times 10^{12}$ cryst./cm^2^ upon introducing 2%, 4%, and 8% Fe-Ni co-doping levels Ni, respectively. 

#### 3.3.4. Crystal Imperfections Studies

**Figure 5:**
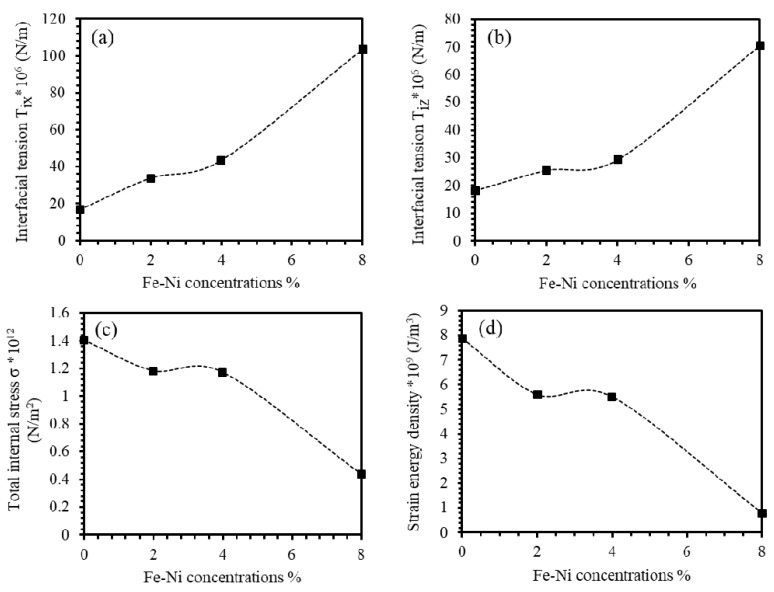
(**a**,**b**) Interfacial tensions, (**c**) total internal stress, and (**d**) strain energy density of undoped ZnO and (Fe-Ni) co-doped ZnO thin films as a function of (Fe-Ni) concentration.

The crystal imperfection defects determine choosing some nanomaterials for definite prospective applications. In-plane and out-plane lattice mismatch result in in-plane and out-plane lattice strains that can be expressed as (Δa/a) and (Δc/c), respectively [49,50]. We found that the behaviors of (Δa/a) and (Δc/c) are nearly identical suggesting the invariance of the unit cell. The tendency of the crystallites of films to have a spherical interface is known as the interfacial tensions along the x-axis and y-axis ($T_{ix}$ and $T_{iz}$), respectively. Solliard and Flueli evaluated the interfacial tensions $(T_{ix}$ and $T_{iz}$) in terms of (N/m) as [22,31,51],

(1) $$T_{ix}=-\frac{3KD}{4}\left(\frac{\Delta a}{a_{0}}\right)$$

(2) $$T_{iz}=-\frac{3KD}{4}\left(\frac{\Delta c}{c_{0}}\right)$$

where K is the bulk modulus and D is the crystallite size. The value of the interfacial tension of ZnO thin film was found to be $16.72\times 10^{6}$ N/m^2^ and $18.10\times 10^{6}$ N/m^2^ for the lattice parameters (a and c), respectively. As indicated in Figure 5a,b, both $T_{ix}$ and $T_{iz}$ increase by increasing Fe-Ni content in the ZnO thin films. Obviously, insertion of 8% of Fe-Ni contents into ZnO thin films increase $T_{ix}$ to 6.18 times that of undoped ZnO thin film. Nonetheless, for the same Fe-Ni concentration, the $T_{iz}$ rises to 3.88 times that of undoped ZnO thin film. The ratio TixTiz is 1.592, which is consistent with the axial ratio *c*/*a* as demonstrated in Figure 2. The internal stresses occur because of many reasons, such as temperature variations, phase transitions, and deformations. The value of the total internal stress (σ) is defined by Young’s Modulus (*E*) of the material and the micro strain 〈ε〉 by the relation σ=E∗〈ε〉. The value of the total internal stress of ZnO thin films was found to be $1.40\times 10^{12}$ N/m^2^. It decreases by introducing Fe-Ni co-doping into ZnO thin films as displayed in Figure 5c. The value of the strain energy density within thin films $\left(E_{d}\right)$ [$J/m^{3}$] depends on the Young’s modulus of the film, the volume of the unit cell (V) and the internal micro strain, 〈ε〉. The strain energy density can be expressed as [44,52],

(3) $$E_{d}=\frac{\sigma^{2}}{2E}=\frac{1}{2}\sigma \langle \varepsilon \rangle =\frac{1}{2}E{\langle \varepsilon \rangle }^{2}$$

where σ and *E* are total internal stress and Young’s modulus. The value of the strain energy density of ZnO thin film was found to be $7.88\times 10^{6}$ N/m. It decreases as Fe-Ni content in ZnO thin films increases as shown in Figure 5d. We found that the parameter *E*_d_ of 8% Fe-Ni ZnO thin films drops to one-tenth of the corresponding value of non-doped ZnO film consistent with the density of dislocations behavior as demonstrated in Figure 4a.

### 3.2. Optical Characterization Analysis 

The optoelectronic characteristics of undoped ZnO and Fe-Ni co-doped ZnO thin films deposited at various Fe-Ni concentrations were examined by analyzing the transmittance T%(λ) and the reflectance R%(λ) spectra in the wavelength range (250–700) nm [53]. Generally, optical properties of semiconducting films are deduced in assessment of the interplay between the incident light and the nanocomposite thin films optical and dielectric constants. The optical properties are sensitive to the preparation technique, preparation conditions, surface morphology, and doping levels [22].

**Figure 6:**
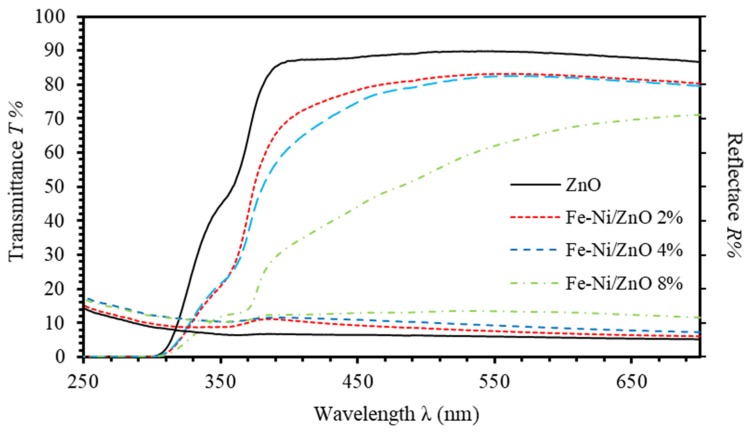
The transmittance and reflectance spectra of undoped ZnO and Fe-Ni co-doped ZnO thin films for various concentrations of Fe-Ni.

Figure 6 shows the transmittance and reflectance spectra of the undoped ZnO and the Fe-Ni co-doped ZnO thin films for various Fe-Ni concentrations. Clearly, as-grown thin films are found to exhibit high transparency in the range (λ≥350 nm, T%≥80%) except for the 8% Fe-Ni co-doped ZnO thin films. The transmittance values of the undoped ZnO thin films in the visible region were found to be about 87%. Introducing Fe-Ni content into ZnO thin films was found to decrease the transmittance values. The 2% and 4% Fe-Ni co-doped ZnO thin films were found to adopt low values of transmittance in the middle of visible range (about 83% and 82%), respectively. We found that 2% Fe-Ni co-doped ZnO thin film are transparent. However, 8% Fe-Ni co-doped ZnO thin films were found to display the lowest transmittance (approximately 60%). Moreover, optical absorption edge is found to shift into the red region upon introducing Fe-Ni contents into ZnO thin films. Therefore, a considerable decrease in band gap energy is achieved. Several reasons can be identified for the observed band gap energy decrease such as, the Fe/Ni-related charge transfer transition in the case of Fe-Ni co-doped ZnO, the enhanced sp-d interactions between the band electrons of ZnO and the localized d electrons of iron and nickel, and the strong mismatch between the electronegativities of Fe, Ni, and Zn in the ZnO system [54,55,56,57,58]. The reflectance was measured by UV-Vis spectrophotometer. Similar interpretation could be applied to the reflectance spectra with reverse behavior to those of the transmittance spectra as demonstrated in Figure 6. We found the reflectance of ZnO thin films to be around 6% when a 500 nm light is incident on the films. Upon introducing Fe-Ni contents into ZnO thin films, reflectance rises to around 13% at 8% of Fe-Ni co-doping level. Past the absorption brink, transmittance and reflectance sum up to unity indicating minimum scattering of the incident light as confirmed by the XRD patterns in Figure 1 [59,60,61].

**Figure 7:**
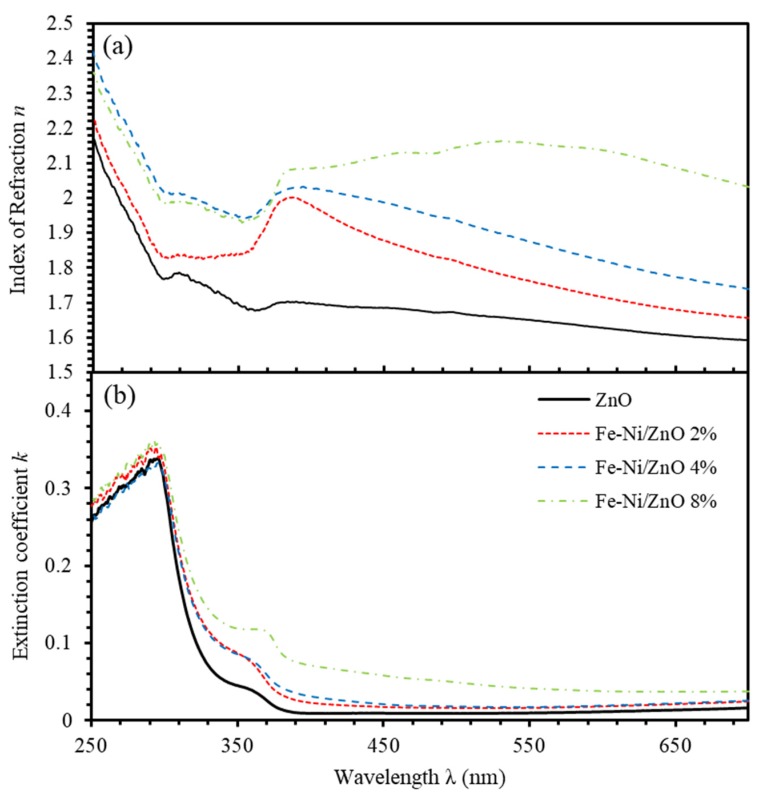
(**a**,**b**) The index of refraction (n) and extinction coefficient (k) of undoped ZnO and (Fe-Ni) co-doped ZnO thin films for different Fe-Ni co-doping levels.

Fabrication and modulation of optical devices such as, optical buttons and filters require full determination of complex refractive index (N=n+ik) and complex dielectric functions ($\varepsilon =\varepsilon_{1}+i\varepsilon_{2}$) [53]. The extinction coefficient (k) and the index of refraction (n) can be expressed as respectively [20],

(4) k=αλ/4π

(5) $$n=\left(1+R/1-R\right)+\sqrt{\left(4R/{\left(1-R\right)}^{2}\right)-k^{2}}$$

where α is the absorption coefficient given by α=(1/d)ln(1/T) and d stands for the thickness of thin film that was found to be 500 nm. The index of refraction (n) as a function of the wavelength of incident light is shown in Figure 7a. The values of n of undoped ZnO thin film assumes values in the range 1.6–1.7. As Fe-Ni content increases, n increases to around 2.1 at 8% Fe-Ni co-doping level. This could be interpreted in terms of the presence of institutional additives as more Fe-Ni content is introduced in the films that increases the reflectance. Moreover, the surge in the refractive index of Fe-Ni co-doped ZnO thin films can be attributed to the solidification of smaller ions into larger collections [62]. Figure 7b shows the extinction coefficient (k) of ZnO and the Fe-Ni co-doped ZnO thin films. The index *k* increases as Fe-Ni concentration increases in the detectable region. It is clearly seen that ZnO film has a minimum constant value (0.02) all over the visible range. However, the Fe-Ni co-doped thin films were found to attain exponentially decreasing trend indicating the presence of Urbach absorption tail. 

**Figure 8:**
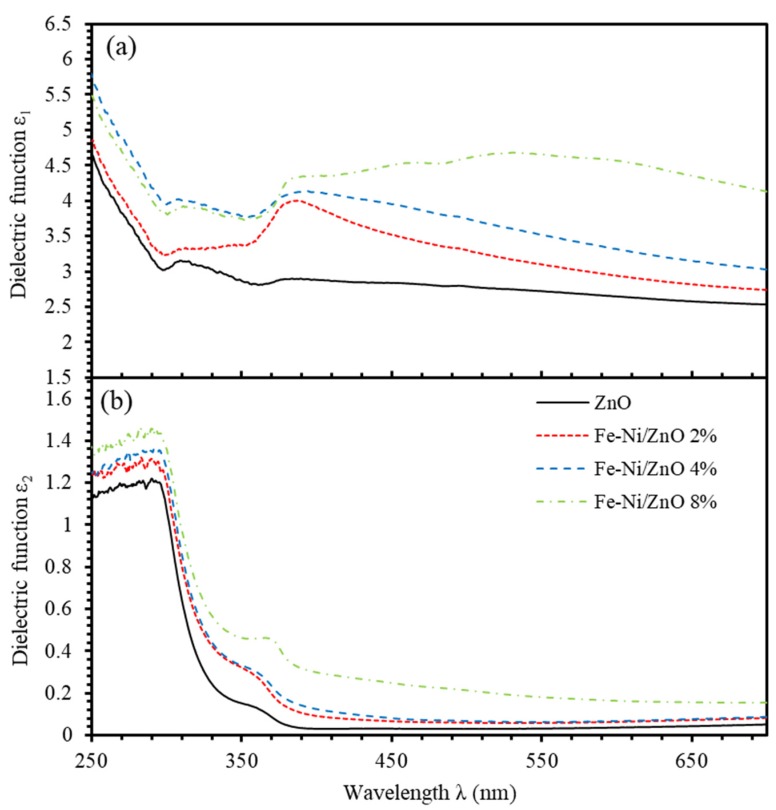
(**a**,**b**) Dielectric functions (ɛ_1_) and (ɛ_2_) of undoped ZnO and Fe-Ni co-doped ZnO thin films for different Fe-Ni co-doping levels.

The complex dielectric function $\left(\varepsilon =\varepsilon_{1}+i\varepsilon_{2}\right)$ is correlated with refractive index (N) by $\varepsilon =N^{2}$. The computation of dielectric function is done in terms of optical constants *n* and *k*, that are related to the real (ɛ_1_) and imaginary (ɛ_2_) parts of the dielectric function by the relations: $\varepsilon^{\prime }=n^{2}-k^{2}$ and $\varepsilon^{\prime \prime }=2nk$ [63]. Figure 8 shows $\varepsilon_{1}$ and $\varepsilon_{2}$ of undoped ZnO and Fe-Ni co-doped ZnO thin films as functions of incident wavelength for different Fe-Ni co-doping levels. Similar argument applies to the complex dielectric function that exhibits a behavior similar to that of the refractive index. We found that, introducing Fe-Ni contents into ZnO thin films increases the dielectric functions.

**Figure 9:**
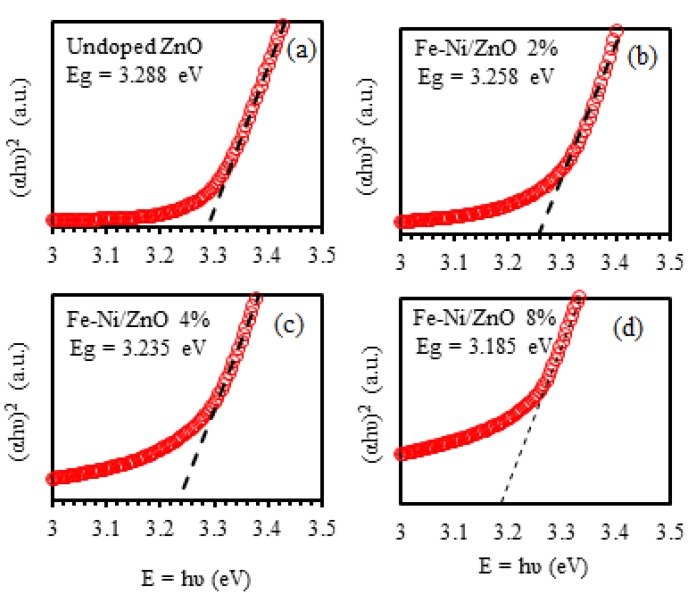
Tauc $E_{g}$ of (**a**) undoped ZnO, (**b**) 2%, (**c**) 4% and (**d**) 8% of Fe-Ni co-doped ZnO thin films.

**Figure 10:**
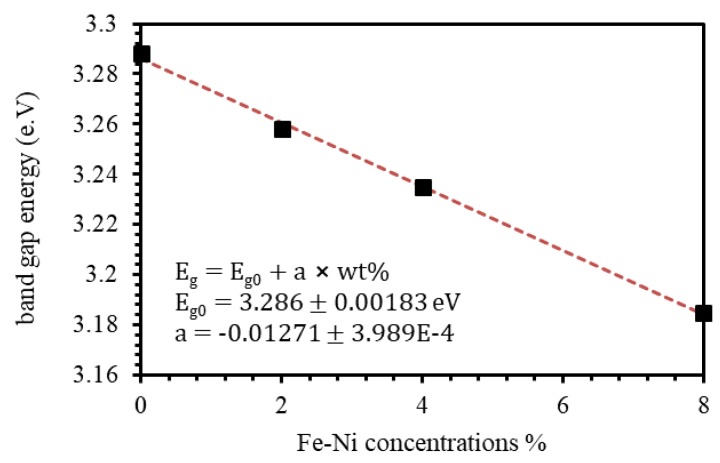
$E_{g}$ of undoped ZnO and Fe-Ni co-doped ZnO of thin films as a function of Fe-Ni co-doping levels.

Tauc plot (hv in e.V.) and ${\left(\alpha h\upsilon \right)}^{2}$ was plotted according to the formula:

(6) $${\left(\alpha hv\right)}^{2}=\beta \left(hv-E_{g}\right)$$

where β is the band trailing parameter and $E_{g}$ is the optical band gap [64]. Figure 9 shows Tauc plot of all investigated thin films. We extrapolate the linear portion of the relationship to intersect the $hv-E_{g}$ axis. The value of $hv-E_{g}$ at which the straight line intersects the axis gives the value of $E_{g}$. The value $E_{g}$ of ZnO film was found to be 3.288 eV. As Fe-Ni is introduced into ZnO films, $E_{g}$ value decreases to 3.258, 3.235, and 3.185 eV for 2%, 4%, and 8% Fe-Ni doping levels, respectively. Figure 10 shows the $E_{g}$ of undoped ZnO and Fe-Ni co-doped ZnO thin films versus Fe-Ni co-doping levels. Our results indicate that the decrease in $E_{g}$ as a function of Fe-Ni co-doping level is linear with a slope of a=−0.01271.

### 3.3. Optoelectronic Parameters

Understanding the reaction of matter to electromagnetic (EM) waves in terahertz (THz) frequency range is extremely important for the functioning of several electromagnetic and optoelectronic devices. EM waves of high frequency interaction with matter has several interesting applications, such as their capability to penetrate wave-guided structures and the ability to transfer broadband information over extended distances. We analyze the refractive index information to get the ratio of free carrier to the effective mass $N/m^{*}$ and the short-wavelength dielectric constant $\varepsilon_{\infty }$ as suggested by Spitzer-Fan [65,66],

(7) $$\varepsilon^{\prime }=\varepsilon_{\infty }-\frac{1}{4\pi^{2}\varepsilon_{0}}\left(\frac{e^{2}}{c^{2}}\right)\left(\frac{N_{c}}{m^{*}}\right)\lambda^{2}$$

where e is the electronic charge, $N_{c}$ is the compactness of states, $m^{*}$ is the effective mass of the charge transporters. Plotting $\varepsilon^{\prime }$ versus $\lambda^{2}$ indicates a linear relationship in the long wavelength zone. It is customary to determine the ratio $N/m^{*}$ from the slope of the straight line, whereas $\varepsilon_{\infty }$ is determined by taking the asymptote of the linear part extended to $\lambda^{2}=0$. The values obtained are presented in Table 1. We found that $\varepsilon_{\infty }$ of ZnO thin films is 2.993 that would surge as Fe-Ni co-doping level is steadily increased. Our results explicitly validate that increasing the number free charge transporters in ZnO and (Fe-Ni) co-doped ZnO thin films strongly contributes to the polarization process [67,68]. Furthermore, fictional part of the dielectric function ($\varepsilon^{\prime \prime }$) in relevance to the wavelength of the incident photon can be analyzed to accurately estimate the relaxation time (τ), optical mobility ($\mu_{opt}$) and optical resistivity ($\rho_{opt}$) by Drude free electron model as given by [65],

(8) $$\varepsilon^{\prime \prime }=\frac{1}{4\pi^{3}\epsilon_{0}}\left(\frac{e^{2}}{c^{3}}\right)\left(\frac{N_{c}}{m^{*}}\right)\left(\frac{1}{\tau }\right)\lambda^{3}$$

The parameter τ can usually be found from the slope of the plot of $\varepsilon^{\prime \prime }$ against $\lambda^{3}$ and from the value of $N_{c}/m^{*}$ by taking $m^{*}=0.44m_{e}$ [69]. Moreover, the plasma frequency $\omega_{p}$ can be expressed as $\omega_{p}=e^{2}N_{c}/m^{*}/\epsilon_{0}\varepsilon_{\infty }$ [44]. The calculated values of $\omega_{p}$ are listed in Table 1. Obviously, $\omega_{p}$-values adopt increased values upon introducing Fe-Ni dopants in ZnO thin films. The greater values of $\omega_{p}$ are correlated with the large values of $N/m^{*}$ and polarization as demonstrated in Table 1. Accurate determination of τ leads to accurate determination of $\mu_{opt}$ and $\rho_{opt}$ of thin films since $\mu_{opt}=e\tau /m^{*}$ and $\rho_{opt}=1/e\mu_{opt}N_{c}$ [65]. The calculated values of optical mobility ($\mu_{opt}$) and optical resistivity ($\rho_{opt}$) are recorded in Table 1. 

## 4. Conclusions

In summary, sol gel dip coating technique was used to synthesize undoped ZnO and the 2%, 4%, and 8% Fe-Ni co-doped ZnO thin films on glass substrate. The Fe-Ni co-doped ZnO thin films are grown by adding gallium nitrate (Ga (No_3_)_2_) to the solution of ZnO for different molar percentages (0%, 2%, 4%, and 8%). The XRD results indicate that undoped ZnO thin film crystallizes in a hexagonal wurtzite structure. The in-plane and out-plane lattice constants (*a* and *c*) are evaluated and found to agree well with those of bulk ZnO. Both constants were found to increase as the Fe-Ni content in ZnO thin films increases. In addition, crystallite size and micro strain are calculated. The value of crystallite size of ZnO was found to be 11.42 nm that increases by incorporating higher Fe-Ni concentrations into ZnO thin films. On the other hand, micro strain was found to decrease as Fe-Ni concentration is increased.

We characterized the optical properties of the as-grown thin films. We measured and characterized the transmittance and reflectance of the undoped and doped ZnO thin films. Furthermore, we investigated the absorption, index of refraction, extinction coefficient, absorption coefficient, and the optical band gap energies as functions of the co-dopants (Fe-Ni) concentration. The transmittance of the undoped ZnO thin film in the visible region was on average >80%. We found that the transmittance decreases as the Fe-Ni concentration in ZnO films increases. The relevant optical properties were observed to be affected accordingly. Therefore, Fe-Ni co-doped thin ZnO films could be used as a key component in a number of optoelectronic devices such as, liquid-crystal displays, OLEDs, smart phones touchscreens, and photovoltaics. Interestingly, we found the index of refraction in the visible region of the undoped ZnO thin film (1.60–1.70) increases significantly as Fe-Ni concentration increases. We found that the index of refraction of Fe-Ni co-doped ZnO thin films assumed an increasing trend and attained a value of 2.20 for 8% Fe-Ni co-doped ZnO thin films. The optical band gap energy of the undoped ZnO thin film was found to be as large as 3.288 eV. Remarkably, optical band gap energy of the Fe-Ni co-doped ZnO thin films decreased linearly as a function of Fe-Ni concentration. Our results on optical band gap of the co-doped thin films anticipated that band gap engineering can be achieved solely by changing the concentration of the Fe-Ni co-dopants. Thus, it is possible to design Fe-Ni co-doped ZnO based devices for specific applications.

## Figures and Tables

**Table 1:** Estimation of some essential optoelectronic constants of ZnO and (Fe-Ni) co-doped ZnO thin films for different (Fe-Ni) co-doping levels.

Parameter	ZnO	Fe-Ni/ZnO 2%	Fe-Ni/ZnO 4%	Fe-Ni/ZnO 8%
Density of states, $N_{c}/m^{*}*10^{+57}$ ($m^{-3}\cdot Kg^{-1}$)	1.208	2.167	2.990	4.008
Charge carrier density, $N_{c}*10^{+26}$ ($m^{-3}$)	4.841	8.687	11.985	16.067
High-frequency dielectric constant, $\varepsilon_{\infty }$	2.993	3.562	4.171	5.703
Relaxation time, $\tau *10^{-14}$ (s)	0.913	1.276	1.657	7.874
Plasma frequency, $\omega_{p}*10^{+7}$	5.720	8.620	10.200	9.960
Optical mobility, $\mu_{opt}*10^{-3}$	3.643	5.095	6.612	31.143
Optical resistivity, $\rho_{opt}*10^{-6}$	3.544	1.412	0.789	0.124
